# Multispectral airborne imagery in the field reveals genetic determinisms of morphological and transpiration traits of an apple tree hybrid population in response to water deficit

**DOI:** 10.1093/jxb/erv355

**Published:** 2015-07-23

**Authors:** Nicolas Virlet, Evelyne Costes, Sébastien Martinez, Jean-Jacques Kelner, Jean-Luc Regnard

**Affiliations:** ^1^Montpellier SupAgro, UMR AGAP 1334, TA-A-108/03, Av. Agropolis, 34398 Montpellier Cedex 5, France; ^2^ Present address: PCBS Department, Rothamsted Research, Harpenden, AL5 2JQ, UK; ^3^INRA, UMR AGAP 1334, TA-A-108/03, Av. Agropolis, 34398 Montpellier Cedex 5, France

**Keywords:** *Malus*×*domestica*, multispectral imagery, quantitative trait locus (QTL), surface temperature, thermal infrared, vegetation index.

## Abstract

Genetic studies of response to water deficit in adult trees are limited by low throughput of the usual phenotyping methods in the field. Here, we aimed at overcoming this bottleneck, applying a new methodology using airborne multispectral imagery and *in planta* measurements to compare a high number of individuals.

An apple tree population, grafted on the same rootstock, was submitted to contrasting summer water regimes over two years. Aerial images acquired in visible, near- and thermal-infrared at three dates each year allowed calculation of vegetation and water stress indices. Tree vigour and fruit production were also assessed. Linear mixed models were built accounting for date and year effects on several variables and including the differential response of genotypes between control and drought conditions.

Broad-sense heritability of most variables was high and 18 quantitative trait loci (QTLs) independent of the dates were detected on nine linkage groups of the consensus apple genetic map. For vegetation and stress indices, QTLs were related to the means, the intra-crown heterogeneity, and differences induced by water regimes. Most QTLs explained 15−20% of variance.

Airborne multispectral imaging proved relevant to acquire simultaneous information on a whole tree population and to decipher genetic determinisms involved in response to water deficit.

## Highlight

This research successfully used image-based spectral indices acquired in the field to assess variability of response to drought in a tree mapping population and to detect the related genetic determinisms.

## Introduction

According to current climate change models for the 21^st^ century, an increase in global mean temperatures is expected, with longer or more frequent episodes of extreme temperatures and drought, notably in the Mediterranean basin (IPCC, 2014). Climate change will lead to reconsideration of bree ding programmes for many crops, and optimization of water use by improving the plant water use efficiency and/or the tolerance to drought will become an increasingly important issue (Hamdy *et al.*, 2003; Condon *et al.*, 2004). Although plant behaviour in response to drought can be analysed in terms of survival (McDowell *et al.*, 2008), it more usually refers to the ability of one genotype to yield better than another under more or less severe water deficit. However, while breeding programmes in fruit species have not yet included drought tolerance among the targeted traits, some authors consider that tree response to water scarcity will become a crucial character to consider (Bassett, 2013).

Plants have developed various mechanisms to cope with drought that depend on the duration and intensity of the water deficit, and their responses occur at different temporal and spatial scales, from cell to whole tree level (Jones *et al.*, 2002). One first response to soil drought is stomatal closure, an avoidance mechanism mediated by the hormone abscisic acid (Pantin *et al.*, 2013). A main consequence of stomatal closure is the decrease in CO_2_ influx and assimilation, which can lead to carbon depletion. When transpiration is reduced by stomatal closure, the outgoing water vapour flux and the latent heat dissipation are also reduced. Stomatal closure thus induces an increase in leaf temperature with a risk of heat stress (Tardieu, 2005). However, efficiency of stomatal regulation is variable according to species and Tardieu and Simonneau (1998) have shown that plants display contrasting transpiration behaviours (isohydric vs anisohydric) in response to drought. At the intra-specific level, genetic variability of stomatal regulation has also been highlighted in apple (Massonnet *et al.*, 2007; Liu *et al.*, 2012) and grapevine (Marguerit *et al.*, 2012; Coupel-Ledru *et al.*, 2014).

As leaf or canopy temperature can be used as a proxy for stomatal conductance, thermal infrared (TIR) imagery appears as a powerful tool to reveal genetic variability of stomatal behaviour (Jones *et al.*, 2009). Numerous indices have been developed to assess crop water stress from canopy surface temperature (*T*_*s*_) with data acquired in signal or imagery mode, from aerial platforms (satellites, aircrafts, unmanned aerial vehicles) or sensors installed directly in the field to observe crop canopies (White *et al.*, 2012). *T*_*s*_ minus air temperature (*T*_*a*_) is a raw variable that is easy to extract from images, but it is sensitive to rapid changes in environmental conditions such as radiative conditions, wind speed and air vapour pressure deficit (Maes and Steppe, 2012).

The presence of mixed soil/plant pixels is a recurring problem when TIR imagery is applied to phenotyping of heterogeneous covers (Hackl *et al.*, 2012; Jones and Sirault, 2014). It is generally considered that using the vegetation surface temperature directly is risky, because the weight of mixed or soil image pixels yielded in porous plant covers can create a shift towards the soil surface temperature (Jackson *et al.*, 1981). To overcome the limitations of environmental and soil influence on *T*_*s*_, Moran *et al.* (1994) developed a Water Deficit Index (*WDI*) based on the Vegetation Index−Temperature (VIT) trapezoid concept, which is applicable to ﬁeld crops with varying contributions of bare soil in the aggregated thermal pixels. This index is particularly suitable for estimation of transpiration rates on heterogeneous vegetation cover. It has been successfully related to the soil moisture and to the plant midday stem water potential (Köksal, 2008; Virlet *et al.*, 2014). Different authors indicated that the use of aerial vectors (ultralight aircraft or unmanned aerial vehicle) coupled with high resolution sensors enables to distinguish the individual trees within a plant grove, even in the TIR waveband where image resolution is low (Berni *et al.*, 2009; Stagakis *et al.*, 2012). Moreover, the intra-crown *T*_*s*_ variability has also been used in tree crops as complementary indicator of moderate water stress effect (González-Dugo *et al.*, 2012), confirming previous work that considered leaf temperature distribution as better indicator of stress than its average (Fuchs, 1990).

Apart from TIR imagery, plant cover can be characterized by different vegetation indices based on the combination of spectral reflectances measured in visible and near-infrared (NIR) wavebands (Zarco-Tejada *et al.*, 2005). These indices can possibly be acquired by broadband commercial sensors (Lebourgeois *et al.*, 2008). In the remotely-sensed image, reflectance in the Red band is affected by light absorption of leaf pigments (mainly chlorophyll a), while the NIR waveband is affected by the scattering in the medium (Zarco-Tejada *et al.*, 2005). Therefore, vegetation indices computed from Red and NIR, such as the normalized difference vegetation index (*NDVI*), can be related to canopy structure and biomass production (Zarco-Tejada *et al.*, 2005) and also considered as indicators of tree vigour. However, *NDVI* is sensitive to low chlorophyll concentration (Peng and Gitelson, 2011) and it also tends to saturation when leaf area index (*LAI*) is higher than 3 or 4. Two other indices only retrieved from visible bands were used: the visible atmospherically resistant index, *VARI*, which shows a better sensitivity to higher values of vegetation cover fraction (Gitelson *et al.*, 2002) and the simple ratio pigment index, *SRPI*, which enables characterization of the crop nitrogen status, being sensitive to change in the pigment relative content (chlorophyll vs carotenoids) (Peñuelas *et al.*, 1994, 1995).

Recent studies on field crops, e.g. wheat (Babar *et al.*, 2006; Comar *et al.*, 2012), maize (Cairns *et al.*, 2012) and cotton (Andrade-Sanchez *et al.*, 2014) assessed potentiality of vegetation indices to be used for large-scale phenotyping. More generally, plant phenotyping based on multispectral or hyperspectral imagery shows promise as a non-invasive method adapted for screening a wide range of individuals in a short period of time. Connecting genotype to phenotype on large datasets currently sustains the development of pheno mics (Furbank and Tester, 2011; Fiorani and Schurr, 2013).

To date, quantitative genetic analyses of tree features in fruit crops have mostly concerned disease resistance, yield and production regularity (Guitton *et al.*, 2012; Celton *et al.*, 2014), and plant architecture (Segura *et al.*, 2008). Owing to low-throughput techniques, few studies on genetic determi ni sms of traits related to water use have been undertaken in these crops except recently in grapevine (Marguerit *et al.*, 2012). Other perennials like forest trees have been compared in natural environments (Brendel *et al.*, 2008) and controlled environments (e.g. *Salix*: Rönnberg-Wästljung *et al.*, 2005; *Populus*: Street *et al.*, 2006) to distinguish well-irrigated and water deficit conditions and to study the genetic/genomic bases of responses to drought and/or water use efficiency.

In this study, we assumed that a genetic analysis could be performed on an apple segregating population submitted to contrasting water regimes, considering different traits mainly issued from airborne multispectral imagery. An experiment was conducted in two successive growing seasons, during which image-based phenotypic variables and agronomic traits such as fruit production or trunk diameter (a proxy for tree vigour) were analysed for both well-watered and water-stress conditions, as well as the difference between the two for a given genotype. For each image-based variable, we considered the mean value of a representative tree crown zone and the variation within this zone, on which mean broad-sense heri tability was computed from genetic linear models. Using a genetic map, quantitative trait loci were detected. Altogether these results demonstrate the relevance of airborne imagery for high-throughput phenotyping of individual trees in the field for their response to water stress and provide the first demonstration that QTL detection could result from such methodology and plant descriptors.

## Materials and methods

### Field experiments and meteorological measurements

The apple tree population studied consisted of progeny derived from a ‘Starkrimson’×’Granny Smith’ cross, characterized by variability in tree vigour, architectural traits (Segura *et al.*, 2008), biennial bearing (Guitton *et al.*, 2012), hydraulic traits (Lauri *et al.*, 2011) and stomatal regulation in response to vapour pressure deficit (Regnard *et al.*, 2009). In February 2007, four replicates of 122 F1-hybrids and their two parents were grafted onto M9 rootstock and randomly planted in an experimental field at the INRA Melgueil experimental station (Diaphen platform, southeast of France, 43°36ʹ N, 03°58ʹ E). Plantation consisted of 10 rows oriented northwest–southeast, with 5×2 m planting distances. The orchard management was performed consistently with professional practices, throughout the study. Automated soil resistivity profiling conducted in March 2009 showed that the soil of the trial plot (at depths of 0−50cm and 50−100cm) could be considered spatially homogeneous for water-holding capacity, and this was confirmed by soil profile descriptions. The field plot was irrigated using a system of microsprayers located in the rows, with one emitter per tree. During summer, contrasting hydric regimes were established. Full irrigation was ensured in half of trees [two replicates per genotype, well-watered trees (WW)], while irrigation was withheld in the other half, resulting in progressive summer soil drought [two replicates per genotype, water-stressed trees (WS)] since the summer rainfall was negligible. Trees submitted to water deficit during summer were the same during the 2010 and 2011 seasons, and three dates per year were studied, representing various water regimes in order to disentangle genotypic and environmental effects in the tree response. Water regimes developed in WW and WS treatments are illustrated by the soil hydric potential mean values (*Ψ*_*soil*_, Table 1A). Micrometeorological data acquired at field included global radiation (*R*_*g*_), air temperature (*T*_*a*_), air relative humidity (*HR*), air vapour pressure deficit (*VPD*), wind speed (*u*) and rainfall (Table 1A).

**Table 1.** (A) Environmental conditions in the field in apple experimental field during image acquisitions in 2010 and 2011: mean values (and SDs) for six dates (see text for detail) *R*_*g*_, global radiation; *T*_*a*_, air temperature; *HR*, air relative humidity; *VPD*, air vapour pressure deficit; *u*, wind speed. Soil hydric potential (*Ψ*_*soil*_): average for six representative well-watered (WW) trees and water-stressed (WS) trees at 30 and 60cm depths. (B) Ultralight aircraft image acquisition system, cameras used and image settings, and original image resolution for each date of experiment.

**A**	**Variables**	**Units**	**Date 1**	**Date 2**	**Date 3**	**Date 4**	**Date 5**	**Date 6**
	Solar time	hh:mm	11:40	10:40	09:50	09:50	10:00	09:20
	*R*_*g*_	W m^-2^	-	782.20 (114.23)	472.83 (33.89)	770.67 (3.27)	599.27 (102.85)	705.00 (0.00)
	*T°*_*air*_	°C	29.72 (0.12)	28.08 (0.42)	23.78 (0.30)	26.91 (0.19)	26.58 (0.33)	26.85 (0.49)
	*HR*	%	44.06 (1.44)	32.97 (1.03)	37.88 (2.57)	58.72 (0.75)	27.96 (0.33)	31.80 (−1.86)
	*VPD*	kPa	2.34 (0.04)	2.55 (0.10)	1.83 (0.11)	1.47 (0.04)	2.51 (0.06)	2.41 (0.14)
	*U*	m s^-1^	2.01 (0.07)	2.72 (0.26)	1.86 (0.10)	1.99 (0.36)	1.73 (0.28)	0.78 (0.32)
	*Ψ*_*soil*_ *WW*	MPa	−0.065 (0.054)	−0.053 (0.028)	−0.066 (0.036)	−0.022 (0.012)	−0.046 (0.039)	−0.024 (0.036)
	*Ψ*_*soil*_ *WS*		−0.099 (0.035)	−0.133 (0.017)	−0.172 (0.022)	−0.031 (0.021)	−0.078 (0.037)	−0.130 (0.048)
**B**			**Date 1**	**Date 2**	**Date 3**	**Date 4**	**Date 5**	**Date 6**
	**Flight altitude**		350 m	330 m	480 m	300 m	300 m	300 m
	**Sensor**							
	RGB		Canon 400D	Canon 500 D	Canon 500 D	Canon 500 D	Canon 500 D	Canon 500 D
	NIR		Canon 400D (+745nm filter)	Canon 500 D (+745nm filter)	Canon 500 D (+745nm filter)	Canon 500 D (+745nm filter)	Canon 500 D (+745nm filter)	Canon 500 D (+745nm filter)
	TIR		FLIR B20HSV	FLIR B20HSV	FLIR B20HSV	FLIR B20HSV	FLIR B20HSV	FLIR B20HSV
	**Setting**							
	RGB	Sensibility	ISO 100	ISO 100	ISO 100	ISO 100	ISO 100	ISO 100
		Shutter speed	1/1250	1/2000	1/2000	1/2000	1/2000	1/2000
		Aperture	F5	F2.8	F2.8	F3.5	F3.5	F3.5
	NIR	Sensibility	100 ASA	100 ASA	100 ASA	ISO 100	ISO 100	ISO 100
		Shutter speed	1/1250	1/2000	1/2000	1/2500	1/2000	1/2000
		Aperture	F5	F2.8	F2.8	F3.5	F3.5	F3.5
	**Initial pixel size (cm**)							
	RGB		5*5	3*3	5*5	3*3	3*3	3*3
	NIR		5*5	3*3	5*5	3*3	3*3	3*3
	TIR		30*30	35*35	53*53	30*30	30*30	30*30
	**Atmospherical correction for TIR image**		No	No	No	Yes	Yes	Yes

### Image acquisitions

The image acquisition system from the ultra-light aircraft consisted of two commercial digital cameras (either Canon EOS 400D or 500D, with 10.1 and 15.1 Megapixel CMOS sensors, respectively, Table 1B) equipped with 35-mm lenses, and one FLIR B20HSV (FLIR Systems Inc., Wilsonville, USA) thermal infrared camera (320*240 matrix) (for details, see: Lebourgeois *et al.*, 2008, 2012; Virlet *et al.*, 2014). One camera acquired visible images in red, green and blue bands (RGB). The second was modified according to Lebourgeois *et al.* (2008, 2012) to obtain images in near-infrared (NIR). Three flights per year were performed during the summers of 2010 and 2011 (Table 1A, 1B). In 2010, flights were realized for low, intermediate and severe water constraints, respectively 8, 27 and 41d after the beginning of drought (Dates 1, 2 and 3). In 2011, the first flight (Date 4) occurred 17 d before the beginning of the drought period, before WW and WS differentiation, while the second and third flights (Dates 5 and 6) were performed respectively 14 and 34d after the beginning of the drought treatment. During the period of water deprivation (i.e. at Dates 1, 2, 3, 5 and 6) WS trees were not irrigated.

### Spectral image preprocessing and indices computation

Image preprocessing was performed with Erdas Imagine 9.3 software (Intergraph Corporation, Huntsville, USA). Procedure of ortho-rectification for RGB and NIR images and radiometric normalization on invariant field targets between dates are fully described in Lebourgeois *et al.* (2008, 2012) and Virlet *et al.* (2014), as well as image geolocation. Thermal infrared images issued from the six acquisition dates were ortho-rectified on the base of both RGB and NIR images and geo-located as well. For each of the six dates, the difference between the surface and air temperature (hereafter referred to as *TsTa*) was obtained by subtracting from each pixel value of the TIR images the air temperature acquired at ground level. Spatial resolution of RGB and NIR images was lowered from initial resolution (c. 3−5cm) to that of TIR image (30cm). From RGB and NIR bands, three vegetation indices were computed: *NDVI*, *VARI* and *SRPI* (Table 2). *NDVI* and *TsTa* were combined to compute the water deficit index (*WDI*) as described in Virlet *et al.* (2014).

**Table 2.** List of phenotypic variables and equations used

Variables	Descriptions	Equations	Related to	References
*NDVI*	Normalized Difference Vegetation Index	(NIR**−**R)/(NIR+R)	Cover fraction, vegetation density	Rouse *et al.*, 1973 Zarco-Tejada *et al.*, 2005
*VARI*	Visible Atmospherical Resistant Index	(G–R)/(G+R)	Cover fraction, biomass production	Peng and Gitelson, 2011
*SRPI*	Simple Ratio Pigment Index	B/R	Nitrogen content, ratio carotenoid/chlorophyll total	Peñuelas *et al.*, 1994, 1995 Lebourgeois *et al.*, 2012
*T_s_T_a_*	Air-surface temperature difference	$\frac{\left(T_{\min}-\;T_{a})-\;(T_{s}-\;T_{a}\right)}{\left(T_{\min}-\;T_{a})-\;(T_{\max}-\;T_{a}\right)}$	Transpiration rate	
*WDI*	Water Deficit Index		Evapotranspiration	Moran *et al.*, 1994 Virlet *et al.*, 2014
*TCSA*	Trunk Cross Sectional Area (mm^2^)	TC^2^/4π	Vigour, growth	
*NbFr*	Fruit number per tree		Fruit biomass production	
*BmFr*	Fruit biomass per tree (kg)		Fruit biomass production	

NIR, near infrared; R, red; B, blue; G, green; *T*_max_ and *T*_min_, maximum and minimum surface temperatures; *T*_s_, surface temperature; *T*_a_, air temperature; TC, trunk circumference, mm.

For each tree, multispectral-based index values were extracted from a 60cm radius buffer zone containing the central upper part of the tree crown. From each buffer (12−16 pixels), mean and standard deviation, SD, were retrieved and considered as two complementary variables characterizing the vegetation response of individuals. As SD characterized the variation occurring inside the buffer zone, it indicated the degree of heterogeneity of the crown structure for the vegetation index and the variability of transpiration rates for the stress indices.

### *In planta* measurements

Trunk circumference (*TC*) of each tree was tape-measured 15cm above the grafting point each year in February. On that basis, the trunk cross-sectional area (*TCSA*) considered as representative of tree vigour was calculated (Table 2). Fruits were harvested each year between 22 August and 2 September before the resumption of irrigation, irrespective of the real maturity picking date (September). The number of fruits per tree and the harvest fresh mass (kg per tree) were determined.

### Data analyses

Statistical analyses were performed using R software v.2.13.2. (R Development Core Team, 2011). For each variable, phenotypic means were computed from each tree for (i) WW and WS conditions confounded, (ii) WW and WS considered separately and (iii) the difference between them, hereafter referred to as the differential index (DI). For example, the phenotypic mean value of *NDVI* indepen dent of water treatment is referred to as *NDVI*, while *sdNDVI_WS* refers to the phenotypic mean value of the SD in the WS condition.

For each variable, two mixed linear models were built. The first one was used to analyse the response of variables in both WW and WS trees. The second one was used to analyse the drought response of each genotype, through the DI obtained. The first mixed linear models included the irrigation modality (M), date (D) or year (Y), which were considered as fixed effects, while the genotype (G), the interactions between genotype and irrigation modality (G×M), and genotype and date or year (G×D or G×Y) were considered as random effects. For each variable, a selection of the best model was performed through minimization of the Bayesian Information Criterion (BIC). For the DI, effects considered in the mixed linear model were the same as mentioned above, but without M and G×M. For each trait, best linear unbiased predictors (Blups) were extracted for estimation of the G effect, which was considered as independent from the irrigation modality and the date (or year) of experimentation and is hereafter referred to as G-Blup. The Blups corresponding to G×M and G×D effects were computed for each irrigation regime (WW- or WS-Blup), and date (or year) considered separately.

For each variable, when G and interaction effects were included, broad-sense heritability of the mean (*h* ^*2*^_*b*_) was estimated as follows:

$$h_{\;b}^{2}=\frac{\sigma_{G}^{2}}{\sigma_{G}^{2}+\frac{\sigma {\;}_{GxM}^{\;2}}{n_{M}}+\frac{\sigma {\;}_{\varepsilon }^{\;2}}{\;n*n_{M}}}$$

where *n* is the number of trees per genotype (two in the present case), and *n*_*M*_ the number of irrigation modalities (two in the present case). When a G×D (or G×Y) effect was included in the model, the denominator also integrated G×D (or G×Y) variances and was divided by the number of dates (six) or years (two). The residual variance $\sigma {\;}_{\varepsilon }^{\;2}$ was divided by the product of the number of trees per genotype and per irrigation modality and the number of dates (or years). This led us to estimate the broad-sense heritability value of the mean of phenotypic values which accounts for the repetitions of each genotype that were present in the experimental plot, according to Gallais (1989). Phenotypic variables were considered heritable if *h* ^*2*^_*b*_ values were greater than 0.2.

### QTL mapping

The QTL analysis was performed using means and Blups extracted per genotype (G-means, G-Blups) for each variable. A consensus genetic map of STK and GS, which integrated 177 SSR and SNP genetic markers, was used for QTL mapping (Guitton *et al.*, 2012). QTL analyses were carried out using MapQTL^®^6.0 (Van Ooijen, 2009). First, a permutation test was performed to determine the loga rithm of the odds (LOD) threshold at which a QTL was declared signiﬁcant, using a genome-wide error rate of 0.05 with 1000 permutations of the data (Van Ooijen, 2009). In a second step, an interval mapping analysis was carried out with a step size of 1 cM, with a LOD score higher than the threshold. Finally, the nearest marker to each QTL peak was selected as a cofactor to perform a multiple QTL mapping (MQM) (Van Ooijen, 2009). Each signiﬁcant QTL was characterized by its LOD score, its percentage of explained phenotypic variation, and its conﬁdence interval (in cM) corresponding to a LOD score drop of 1 or 2 on either side of the likelihood peak. QTLs that showed clearly overlapping confidence intervals, close LOD peaks and similar allelic effects, were considered to co-localize.

When a QTL was detected with at least two cofactors, models considering markers and their interactions as cofactors were cons tructed using a backward procedure under R software v2.13.2. Models were selected based on the minimum Akaike Information Criterion values (AIC). In the selected model, the global percentage of phenotypic variation (global R^2^) was then estimated. When one marker was derived from only one of the parents, the nearest maker included in the QTL and deriving from both parents was chosen. The location of QTLs on the genetic was finally illustrated using MapChart® (Voorrips, 2001).

## Results

### Variance analysis and heritability

Models selected for vegetation indices were similar whether means or SDs were considered (Table 3A). All vegetation indices were significantly impacted by G, D and M effects. For *NDVI,* the model included only the G×M interaction, whereas for *VARI*, *SRPI*, *TsTa* and *WDI* variables G×M and G×D interactions were also taken into account. Concerning tree vigour and fruit production, the models selected included G and Y effects. For *TCSA* only the G×M interaction was retained in the mixed linear model, while the G×Y interaction was retained for fruit number (*NbFr*) and both G×M and G×Y interactions were retained for fruit yield biomass (*BmFr*). For all DI variables (Table 3B) the models selected included G and D (or Y) effects. G×D was also included for *sdVARI_DI*, *sdSRPI_DI*, *sdTsTa_DI* while G×Y was included for *NbFr_DI* only. It is noticeable that the random interaction effects (G×M and/or G×D) were generally lower than the G effects.

**Table 3.** Description of fixed (M, modality; D, date; Y, year) and random (G, genotype) effects used in selected mixed linear models For each variable, models related to phenotypic values in (A) WW and WS, and (B) models related to DI (differential index: difference of the variable between WS and WW trees) were built. Percentage variances of each random effect and of the residuals (Res), and broad-sense heritability values (*h* ^*2*^_*b*_) are indicated.

**A**		**Fixed effect**			**Random effect**				**% variances**					
		**M**	**D**	**Y**	**G**	**G×M**	**G×D**	**G×Y**	**G**	**G×M**	**G×D**	**G×Y**	**Res**	***h*** ^***2***^_***b***_
	*NDVI*	x	x	-	x	x	-	-	35	19	-	-	46	0.62
	*sdNDVI*	x	x	-	x	x	-	-	21	8	-	-	71	0.50
	*VARI*	x	x	-	x	x	x	-	23	6	9	-	61	0.77
	*sdVARI*	x	x	-	x	x	x	-	9	5	10	-	76	0.56
	*SRPI*	x	x	-	x	x	x	-	19	18	4	-	59	0.60
	*sdSRPI*	x	x	-	x	x	x	-	17	8	5	-	70	0.69
	*TsTa*	x	x	-	x	x	x	-	15	18	5	-	62	0.55
	*sdTsTa*	x	x	-	x	x	x	-	7	9	4	-	79	0.49
	*WDI*	x	x	-	x	x	x	-	11	7	5	-	76	0.59
	*sdWDI*	x	x	-	x	x	x	-	3	6	6	-	85	0.31
	*TCSA*	x	-	x	x	x	-	-	51	15	-	-	34	0.76
	*NbFr*	x	-	x	x	-	-	x	25	-	-	37	37	0.52
	*BmFr*	x	-	x	x	x	-	x	12	8	-	32	49	0.31
**B**		**Fixed effect**			**Random effect**				**% variances**`					
		**M**	**D**	**Y**	**G**	**G×M**	**G×D**	**G×Y**	**G**	**G×M**	**G×D**	**G×Y**	**Res**	***h*** ^***2***^ ***_b_***
	*NDVI_DI*	-	x	-	x	-	-	-	44	-	-	-	56	0.61
	*sdNDVI_DI*	-	x	-	x	-	-	-	19	-	-	-	81	0.32
	*VARI_DI*	-	x	-	x	-	-	-	19	-	-	-	81	0.33
	*sdVARI_DI*	-	x	-	x	-	x	-	13	-	8	-	79	0.62
	*SRPI_DI*	-	x	-	x	-	-	-	35	-	-	-	65	0.52
	*sdSRPI_DI*	-	x	-	x	-	x	-	17	-	7	-	75	0.70
	*TsTa_DI*	-	x	-	x	-	-	-	34	-	-	-	66	0.50
	*sdTsTa_DI*	-	x	-	x	-	x	-	19	-	14	-	67	0.70
	*WDI_DI*	-	x	-	x	-	-	-	17	-	-	-	83	0.29
	*sdWDI_DI*	-	x	-	x	-	-	-	9	-	-	-	91	0.17
	*TCSA_DI*	-	-	x	x	-	-	-	35	-	-	-	65	0.52
	*NbFr_DI*	-	-	x	x	-	-	x	13	-	-	1	86	0.38
	*BmFr_DI*	-	-	x	x	-	-	-	17	-	-	-	83	0.29

Broad-sense heritability *h* ^*2*^_*b*_ for both WW and WS (Table 3A) showed medium to high values (0.49 to 0.77) except for *sdWDI* and *BmFr*, whose heritability was low (0.31). For DI variables (Table 3B), fairly high *h* ^*2*^_*b*_ values (0.50 to 0.70) were found for *NDVI*, *sdVARI*, *SRPI*, *sdSRPI, TsTa, sdTsTa* and *TCSA*. In contrary, *h* ^*2*^_*b*_ for the other variables, including *WDI_DI*, was much lower (0.17 to 0.38) than that found for WW and WS. Moreover, higher *h* ^*2*^_*b*_ were found in *VARI_DI*, *SRPI_DI*, and *TsTa_DI* for SD values than for mean values.

### Correlations between variables

High pairwise positive correlations were observed between *NDVI*, *VARI* and *SRPI* for the G-mean, WW, WS and for DI (Pearson’s *r* from 0.45 to 0.88, Table 4) even though lower *r* values were found between *VARI* and *SRPI* for G-mean and WW (Tables 4A, 4B). These three variables were significantly and negatively correlated with *sdNDVI* and *sdSRPI* for the G-mean, WW, WS and for DI (from −0.46 to −0.66, Table 4), despite much lower correlation being found between *sdNDVI* and these variables for DI (from −0.06 to −0.32, Table 4D). Moreover, *sdNDVI*, *sdVARI* and *sdSRPI* presented pairwise positive correlations for the G-mean and WW (0.33 to 0.88, Table 4A, 4B). For WS and DI, only *sdSRPI* was significantly correlated with *sdNDVI* and *sdVARI* (0.74 and 0.42 respectively, Table 4C, 4D). *WDI* was highly and positively correlated with *sdWDI* (0.49 to 0.52). The trunk diameter variable, *TCSA*, presented generally moderate to high positive correlations with *NDVI*, *VARI* and *SRPI*. The highest correlation was observed with *NDVI*, either for the G-mean, WW, WS or for DI (0.55 to 0.67). Variables relative to fruit production, *NbFr* and *BmFr*, were highly intercorrelated (0.75 to 0.85), and a high positive correlation of these variables with *TCSA* was also observed, particularly for *BmFr* (from 0.50 to 0.55). Moreover, *BmFr* was positively and more highly correlated to *NDVI*, *VARI* and *SRPI* (0.23 to 0.52) than *NbFr* (0.13 to 0.32) for the G-mean, WW and WS.

**Table 4.** Genetic Pearson’s *r* correlations between *NDVI*, *VARI*, *SRPI*, *WDI* variables, and *TCSA*, *NbFr* and *BmFr*, (A) for genetic means of two water regimes confounded, (B) for well-watered trees, (C) for water-stressed trees and (D) for differential index DI. *r* values in bold type were significant for *P*<0.001

	*NDVI*	*VARI*	*SRPI*	*sdNDVI*	*sdVARI*	*sdSRPI*	*WDI*	*sdWDI*	*TCSA*	*NbFr*	*BmFr*		*NDVI*	*VARI*	*SRPI*	*sdNDVI*	*sdVARI*	*sdSRPI*	*WDI*	*sdWDI*	*TCSA*	*NbFr*	*BmFr*
**A**												**B**											
*NDVI*	1												1										
*VARI*	**0.61**	1											**0.74**	1									
*SRPI*	**0.80**	0.28	1										**0.64**	0.26	1								
*sdNDVI*	**−0.58**	**−0.55**	**−0.49**	1									**−0.66**	**−0.60**	**−0.43**	1							
*sdVARI*	0.03	0.15	0.00	**0.36**	1								**−**0.16	**−0.31**	**−**0.13	**0.42**	1						
*sdSRPI*	**−0.64**	**−0.62**	**−0.54**	**0.88**	**0.33**	1							**−0.46**	**−0.46**	**−0.52**	**0.66**	**0.57**	1					
*WDI*	0.15	0.06	0.02	0.16	**−**0.16	0.12	1						**−**0.03	0.21	**−**0.25	0.10	**−0.30**	0.02	1				
*sdWDI*	0.03	0.06	**−**0.14	**0.39**	0.21	0.27	**0.52**	1					**−**0.07	0.01	**−**0.22	0.08	**−**0.11	**−**0.01	**0.52**	1			
*TCSA*	**0.67**	**0.33**	**0.57**	**−0.36**	0.03	**-0.35**	0.13	0.09	1				**0.63**	0.29	**0.45**	**−0.33**	0.01	**−**0.28	0.14	0.07	1		
*NbFr*	**0.32**	**0.32**	0.25	**−**0.28	0.20	**−**0.24	**−**0.21	**−**0.12	**0.39**	1			0.29	0.28	0.13	**−**0.27	0.13	**−**0.14	**−**0.06	**−**0.06	**0.39**	1	
*BmFr*	**0.40**	**0.35**	**0.32**	**−**0.27	0.22	**−**0.23	**−**0.20	**−**0.04	**0.50**	**0.85**	1		**0.48**	**0.38**	0.23	**−0.37**	**−**0.01	**−**0.13	**−**0.03	**−**0.04	**0.50**	**0.85**	1
**C**												**D**											
*NDVI*	1												1										
*VARI*	**0.71**	1											**0.74**	1									
*SRPI*	**0.88**	**0.51**	1										**0.82**	**0.45**	1								
*sdNDVI*	**−0.64**	**−0.53**	**−0.58**	1									**−**0.19	**−**0.06	**−0.32**	1							
*sdVARI*	0.06	-0.10	0.10	0.25	1								**−**0.05	**−**0.29	0.02	**−**0.12	1						
*sdSRPI*	**−0.58**	**−0.60**	**−0.60**	**0.74**	0.25	1							**−0.51**	**−0.52**	**−0.57**	0.25	**0.42**	1					
*WDI*	**−**0.15	0.10	**−**0.12	0.23	**−0.30**	0.10	1						**−**0.16	0.18	**−**0.20	0.07	**−0.34**	0.03	1				
*sdWDI*	**−**0.05	0.04	**−**0.06	**0.35**	0.07	0.17	**0.49**	1					**−**0.09	0.01	**−**0.10	0.06	**−**0.12	0.01	**0.51**	1			
*TCSA*	**0.60**	**0.31**	**0.54**	**−0.31**	0.05	**−0.34**	0.08	0.07	1				**0.55**	**0.48**	**0.30**	0.13	**−**0.06	**−**0.26	**−**0.13	**−**0.10	1		
*NbFr*	0.29	0.29	0.27	**−**0.23	0.22	**−**0.26	**−**0.28	**−**0.13	**0.39**	1			**0.55**	**0.49**	**0.33**	0.22	**−**0.08	**−**0.19	**−**0.03	**−**0.04	**0.52**	1	
*BmFr*	**0.52**	**0.38**	**0.39**	**−**0.23	0.03	**−**0.10	**−**0.21	0.01	**0.50**	**0.85**	1		**0.48**	**0.35**	0.27	0.29	**−**0.04	**−**0.07	**−**0.08	**−**0.01	**0.55**	**0.75**	1

### QTL detection

**Fig. 1.**
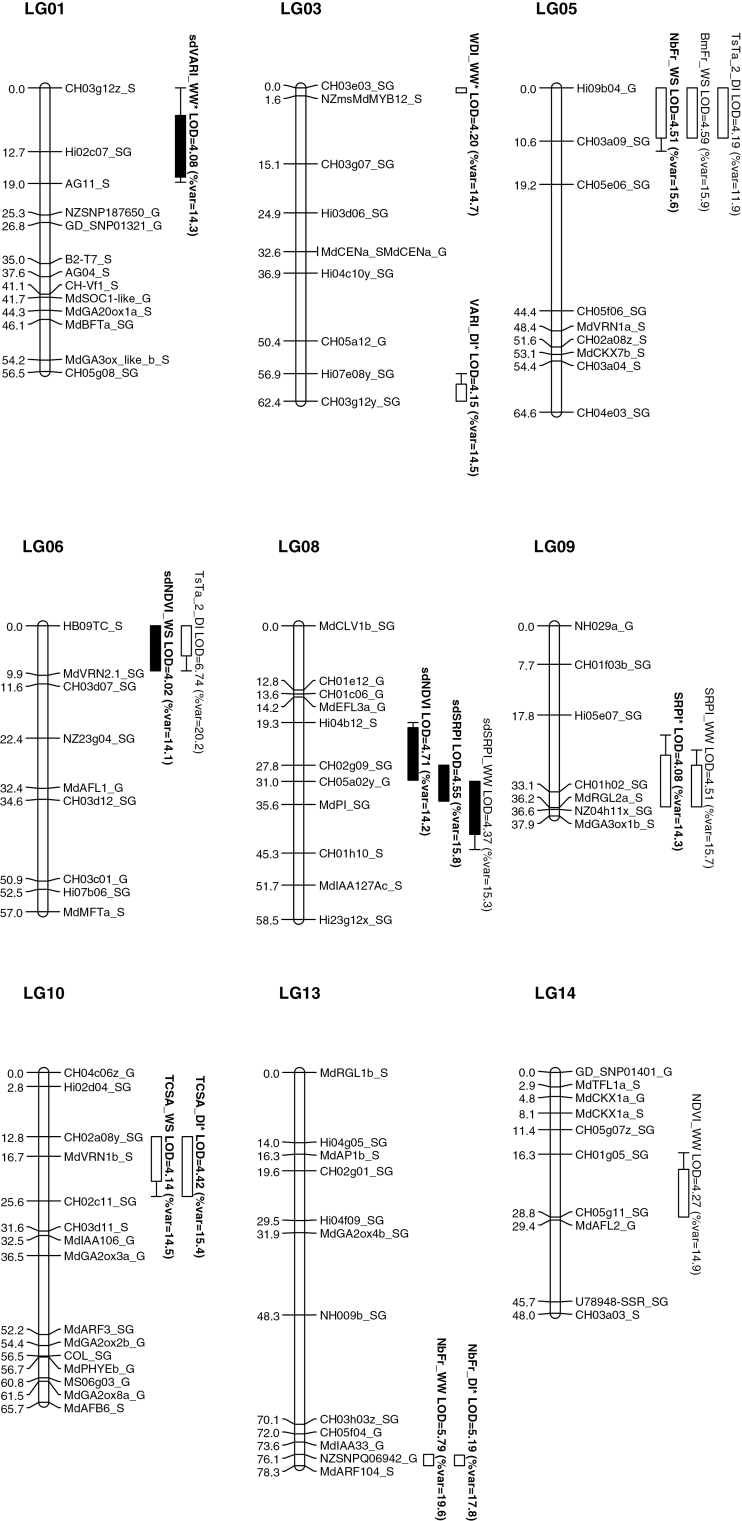
Genomic positions of the QTLs detected on the consensus ‘Starkrimson’×’Granny Smith’ (STK×GS) map. QTLs are represented by boxes, in which length represents the LOD-1 conﬁdence interval and extended lines represent the LOD-2 conﬁdence interval. Boxes relative to QTLs for mean values of variables are in white, and those relative to QTLs for standard deviations SD are in black. QTL detected for G-Blups are in bold type and * stand for QTLs detected for G-Blups and G-means.

Seventy-four QTLs were detected, mapping over 16 of the 17 linkage groups (LGs) of the consensus STK×GS genetic map. As 56 of these QTLs were found only at specific dates, they are not detailed in the following text. The complete list of QTLs detected is presented in Supplementary Table S1 and Supplementary Fig. S1. The results exposed hereafter (Fig. 1; Table 5) are focusing on the 18 most reliable QTLs that were mapped over nine LGs. These QTLs were detected for G-Blup or for the G-mean, and in some cases for both, whatever the date.

**Table 5.** Main QTLs detected on the consensus STK×GS map by multiple QTL mapping (MQM) for variables *NDVI*, *VARI*, *SRPI*, *T_s_T_a_*, *WDI*, *TCSA*, *NbFr* and *BmFr* in well-watered (WW) and/or water-stress (WS) conditions and for the differential index DI (WS−WW) QTLs detected for G-Blups are in bold type and * stand for QTLs detected for G-Blups and G-means.

**Traits**	**LG** ^a^	**LOD** ^b^	**R** ^**2**^ ^c^	**R** ^**2**^ **global** ^d^	**Position**	**Cofactor**	**Allelic effect** ^e^	**Af**	**Am**	**D**
*NDVI_WW*	14	4.27	0.149		26.318	CH05g11_SG	D, Af, Am	-7.80E-03	1.96E-03	-9.11E-03
***sdNDVI***	08	4.71	0.142		29.763	CH02g09_SG	Af	3.15E-03	1.60E-04	-9.93E-04
***sdNDVI_WS***	06	4.02	0.141		1	HB09TC_S	Af	-1.33E-03	3.08E-04	9.00E-05
sdVARI_WW*	01	4.08	0.143		12.749	Hi02c07_SG	D	5.00E-05	-1.75E-06	-1.45E-04
VARI_DI*	03	4.15	0.145		62.417	CH03g12y_SG	D, Af, Am	5.71E-04	-3.94E-04	-6.76E-04
SRPI*	09	4.08	0.143		34.112	CH01h02_SG	Af, Am, D	2.23E-03	1.97E-03	1.86E-03
*SRPI_WW*	09	4.51	0.157		34.112	CH01h02_SG	Am, Af, D	3.14E-03	3.32E-03	1.32E-03
***sdSRPI***	08	4.55	0.158		29.763	CH05a02y_G	Af	1.74E-03	-9.55E-05	-9.76E-04
*sdSRPI_WW*	08	4.37	0.153		35.571	MdPI_SG	Af	3.51E-03	9.96E-04	1.49E-04
*TsTa_2_DI*	05	4.19	0.119		0	Hi09b04_G	Am, Af	-1.48E-01	-1.64E-01	-5.78E-02
	06	6.74	0.202	0.208	0	HB09TC_S	Af, Am	-2.47E-01	1.63E-01	-7.77E-02
WDI_WW*	03	4.2	0.147		0	CH03e03_SG	Am, Af	3.89E-03	-4.50E-03	3.80E-05
***TCSA_WS***	10	4.14	0.145		15.783	MdVRN1b_S	Af, D	-7.03E+01	4.05E+00	2.90E+01
***NbFr_WW***	13	5.79	0.196		77.149	MdARF104_S	Af, Am	-1.07E+01	-1.03E+01	4.52E+00
***NbFr_WS***	05	4.51	0.156		0	Hi09b04_G	Af, D	2.31E+00	-5.11E-01	-1.19E+00
*BmFr_WS*	05	4.59	0.159		0	Hi09b04_G	Af, D	1.89E+00	-4.14E-01	-1.19E+00
TCSA_DI*	10	4.42	0.154		16.687	MdVRN1b_S	Af	-1.57E+02	-1.17E+01	6.40E+01
NbFr_DI*	13	5.19	0.178		77.149	MdARF104_S	Af, Am	2.65E+01	2.55E+01	-8.87E+00

^a^ Linkage group.

^b^ Maximum LOD score value.

^c^ Percentage of phenotypic variation explained by the QTL.

^d^ Percentage of phenotypic variation explained by QTL when it was detected with at least 2 cofactors.

^e^ Female (Af) and male (Am) additive effect estimated as [(μ_ac_+μ_ad_)–(μ_bc_+μ_bd_)]/4 and [(μ_ac_+μ_bc_)–(μ_ad_+μ_bd_)]/4 respectively; dominance (D) estimated as = [(μ_ac_+μ_bd_)–(μ_ad_+μ_bc_)]/4, where μ_ac_, μ_bc_, μ_ad_, and μ_bd_ are the estimated phenotypic means associated with each of the four possible genotypic classes ac, bc, ad and bd, deriving from an <ab×cd> cross.

### QTLs for traits related to vegetation indices NDVI, VARI and SRPI.

For G-Blup of *NDVI* and *SRPI* variables, three QTLs were detected independently of the environment (water regime or date): two QTLs concerned *sdNDVI* and *sdSRPI* and presented a common zone located on LG08. They explained 14.2% and 15.8% of the variability respectively, and were both characterized by female allelic effects. One QTL for *SRPI* was detected on LG09 and explained 14.3% of the variability. It showed female, male and dominance effects.

Four QTLs were detected for specific G-means of WW on four different LGs. Two of these QTLs, related to *NDVI_WW* and *SRPI_WW*, were detected on LG14 and LG09, respectively. They explained 14.9% and 15.7% of the varia bility, and both of them resulted both from female, male and dominance effects. Two other QTLs, related to *sdVARI_WW* and *sdSRPI_WW*, were mapped on LG01 and LG08, and resulted from dominance and female effects, respectively. The QTL for *sdVARI_WW* was also identified for G-Blup.

For WS, one QTL was identified for *sdNDVI_WS* at the top of LG06. It explained 14.1% of the variability and mainly resulted from female effects. For DI, one QTL was detected for *VARI_DI* and mapped at the bottom of LG03 for both G-Blup and G-mean. It explained 14.5% of the variability and resulted from female, male and dominance effects.

#### QTLs for traits related to tree foliage transpiration

One QTL was detected for *WDI* in WW condition, at the top of LG03 for both G-Blup and G-mean. It explained 14.7% of the variability and was characterized mainly by female and male effects. Finally, two QTLs were detected for *TsTa_2_DI* (at Date 2) on LG05 and LG06, respectively. The global linear model indicated an interaction between these two QTLs, which together explained 20.8% of the variability. They mainly resulted from female and male effects. The QTL which mapped on LG06 co-localized with the QTL for *sdNDVI_WS*.

#### QTLs for traits related to tree vigour and fruit production

Two QTLs were detected in relation to the tree vigour (*TCSA*), the first one for *TCSA_WS*, and the second one for *TCSA_DI*. Both mapped on LG10 and co-localized near the MdVRN1b_S marker. These QTLs explained 14.5% and 15.4% of the variability, respectively, and mostly resulted from female allelic effects. For fruit number, one QTL was detected for WW (*NbFr_WW*) at the bottom of LG13. It explained 19.6% of the variability. Another QTL was detected for *NbFr_DI* at the same position, explaining 17.8% of variability. These two QTLs mainly resulted from male and female allelic effects. Finally, two QTLs were found at the top of LG05 for *NbFr_WS* and *BmFr_WS*. They explained 15.6% and 15.9% of the variability, respectively, and both resulted from female and dominance effects. Interestingly, these two QTLs co-localized with the QTL identified for *TsTa_2_DI*.

## Discussion

### Variability of the phenotypic traits

Due to, on the one hand, the changes in environmental conditions and imagery flight parameters and, on the other, the difficulty to apply comparable water constraints from one year to the next, the analysis of the population behaviour undertaken through linear mixed models did take into account the large variability of phenotypic traits (Supplementary Table S2). Among vegetation indices, *NDVI* appeared the most stable vegetation index, independent of the environment and acquisition conditions (no G×D interaction) whereas other spectral indices chosen proved to be sensitive to drought, with a G×D interaction revealed for *VARI*, *SRPI*, *TsTa* and *WDI*. By contrast, the absence of G×D interaction for G-means for the DI variables suggested that the use of the difference between water-stressed and well-irrigated trees somewhat smoothed out the inter-date variations.

To our knowledge, this study is the first one to make use of spectral indices to assess genetic variability in perennial plants in response to drought, and to analyse the related determinisms. As a consequence, comparisons in the ensuing discussion are often referring to results obtained in annual crops. In the present study, the vegetation indices used were extracted from a buffer zone of the same size located in the central zone of each tree crown. As such, the value of these vegetation indices must be considered as more related to the vegetation cover fraction and biomass production rather than to the foliage physiological properties. Whatever the traits considered, either relative to vegetation or to transpiration, moderate to high values of broad-sense heritability were found, indicating an interesting contribution of multispectral imagery for genetic analysis of these traits in a tree population. Concerning the vegetation indices, our results were consistent with those found in annual crops, where high heritability values were also found (e.g. in wheat: Babar *et al.*, 2006; in cotton: Andrade-Sanchez *et al.*, 2014). Heritability values found for agronomic traits in apple, *TCSA*, *NbFr* and *BmFr*, were in the same order of magnitude as those for spectral indices. However, *TCSA,* which is an integrative variable, exhibited the highest *h^2^_b_* value whereas the lower values for *NbFr* and *BmFr* likely resulted from the influence of Y, and Y and M effects, respectively. When heritability was computed for DIs, values of most variables stayed high except for *WDI_DI*, probably because of the composite nature and complexity of this trait. Concerning *TsTa* and *TsTa_DI*, high heritability values were found, consistent with previous results found in wheat (Mason *et al.*, 2013; Rebetzke *et al.*, 2013).

### Trait correlations, QTL detection and co-localization

Positive correlations between the vegetation indices, *TCSA* and fruit production were observed. In particular *NDVI* exhibited the highest correlation with the trunk diameter, *TCSA*, and with fruit yield biomass, *BmFr*. As well as *NDVI*, *TCSA* is generally related to plant size, leaf area and light interception (González-Talice *et al.*, 2012), which suggests that *NDVI* is a good indicator of vigour and development in apple trees. However, QTLs that were found for vegetation indices, fruit yield and *TCSA* did not co-localize, and this indicated that genetic determinisms controlling these traits likely differ. Moreover, while the three vegetation indices presented high and positive intercorrelations, related QTLs did not co-localize. Indeed, QTLs for *NDVI* and *SRPI* were mostly detected on two different LGs: LG14 and LG09, respectively. This could be explained by the spectral bands used: the former vegetation index, making use of NIR for computation, is more related to canopy structure than the second one, only computed from visible bands that are more related to light absorption (Zarco-Tejada *et al.*, 2005; Lebourgeois *et al.*, 2012). Otherwise, the QTL found for *VARI_DI*, which highlights differences between WS and WW tree responses, suggests that drought could affect the radiation absorption capacity, by lowering the fractional vegetation cover.

Intra-crown variations of vegetation indices, *sdNDVI* and *sdSRPI*, presented high and negative correlations with means of *NDVI* and *SRPI,* and moderate and negative correlations with *TCSA*. This indicates that the highest density of vegetation was less variable than the lowest, but also that the vegetation heterogeneity was lower where tree vigour increased. As the intra-crown variability could be an indicator of branching patterns or leaf clumpiness (Da Silva *et al.*, 2014), this needs further investigation. Similarly, as *WDI* and *sdWDI* were posi tively correlated, this could be due to spatial heterogeneity in stomatal conductance within the tree crown in response to moderate stress (González-Dugo *et al.*, 2012). The QTL detected for *sdNDVI_WS* could be attributed to the variation of leaf rolling over genotypes in response to drought, a phenomenon also observed in other species and limiting plant cover fraction (e.g. maize: Lu *et al.*, 2012). Furthermore, *sdNDVI* and *sdSRPI* were strongly intercorrelated and QTLs for these variables co-localized at the middle of the LG08 (Table 5; Supplementary Table S2). The location of these QTLs also matched with a QTL zone for traits involved in gas exchange, xylem conductance and fruit production on the STK×GS population (Regnard *et al.*, 2009; Lauri *et al.*, 2011, Guitton *et al.*, 2012). These co-localizations could be explained by an increased capacity of the plant to transport water, carbohydrates and sugar to the growing organs, as suggested by Lauri *et al.* (2011). Nonetheless, these co-localizations might also be explained by a pleiotropic effect of these QTLs, or by clustering of functionally related genes (Cai and Morishima, 2002). Gene clusters have already been reported in apple for various traits such as resistance to pathogens (Xu and Korban 2002; Baldi *et al.*, 2004). However, discrimina ting between linked and pleiotropic QTLs was not practicable in the present study, considering the limited population size and the density of the genetic map available.

Among QTLs detected for fruit production, two of them, *NbFr_WW* and *NbFr_DI*, were located at the bottom of LG13. This zone was adjacent to the one found for biennial bearing on the same STK×GS population (Guitton *et al.*, 2012). Otherwise, a year-specific QTL, *NbFr_11*, was detected at the same location as *NbFr_WW* and *NbFr_DI* (Supplementary Fig. S2), which could confirm the importance of this zone in the control of biennial bearing. In addition, QTLs for fruit production in WS trees (*NbFr_WS* and *BmFr_WS*) and for leaf temperature (*TsTa_2_DI*) co-localized on LG05. Although matching for those traits was only temporary, i.e. when the difference between WW and WS treatments was considered at Date 2 for *TsTa*, it illustrates the negative relationship between yield and leaf temperature. Indeed, as stated by Naor and Girona (2012), a positive link between plant yield and evapotranspiration is generally observed, and the increase in leaf temperature (here in WS trees) is an indicator of lower transpiration rate and likely of lower carbon assimilation. Reduction of stomatal conductance in WS trees could likely be invoked for limitation of fruit production in this case because water constraint was severe (no irrigation occurred during the summer period). Such a causal relation between water withholding and its effect on yield reduction is nevertheless not straightforward in fruit trees (Bassett, 2013), particularly when a moderate water deficit occurs during stages of low fruit growth (Goodwin and Boland, 2002).

Although one QTL was detected on LG03 for *WDI_WW*, no co-localization with fruit production variables was observed. Moreover, genetic correlations of WDI with fruit production variables during the two years of study (Table 4) were not significant, whereas significant negative correlations of this variable were observed with *NbFr* and *BmFr* (*r* value of −0.53 and −0.55 respectively, data not shown) when only year 2011 was considered. This negative correlation in a specific year could be attributed to the propensity to biennial fruit-bearing already shown on this progeny (Guitton *et al.*, 2012).

It has been recently shown that wild germplasm of *Malus* is exhibiting a certain range of tolerance to drought (Bassett, 2013), but available information on the commercial parental genotypes used in the present study is scarce. González-Talice *et al.* (2012) suggested that the ‘Granny Smith’ cultivar has a lower hydraulic conductance and/or stomatal conduc tance than that of the ‘Gala’ cultivar, indicating a stronger sensitivity of the former to high evaporative demand and/or soil drought. In contrast, the ‘Starkrimson’ cultivar is well known for its photosynthetic efficiency (Yang and Wang, 1993), while its response to drought is not documented. In the present study, prevailing female allelic effects were found for almost all variables, suggesting a larger polymorphism and allelic contrast in the ‘Starkrimson’ parent than in ‘Granny Smith’. ‘Starkrimson’ could thus provide interesting alleles for adaptation to drought, even though other *Malus* genetic backgrounds need to be explored in the next future.

Partial homology between LGs that has been described in the ‘Golden Delicious’ apple genome (Velasco *et al.*, 2010) led us to examine homologous regions in which main QTL zones were detected. The median zone on LG08, around CH02g09 in which many QTLs were detected, matches the top of LG15, above CH03b6, where a QTL was detected for *SRPI_1_DI*. Similarly, the QTL zone on the top of LG03 detected for *NDVI_4_WW* corresponds to the region on LG11 on which a QTL was detected for *sdNDVI_1_DI*. By contrast, the QTL zones found in LG05 and LG10 for *NbFr_WS* and *NbFr_11_DI*, respectively, were located on chromosomal fragments that are inverted on their respective chromosome and therefore did not matched. Similarly, the QTLs detected on LG06 that were located either above or below CH03d07 were compared to those detected on LG14, without finding evident homology in those regions. These findings suggest that further investigations of QTL zones homologies will be required, along with identification of candidate genes in the zones of highest interest.

To summarize, this work is an important step in the study of tree field phenotyping for response to abiotic stress. It confirmed—if proof was needed—the strong potential of remote sensing tools as a method for screening a large panel of genotypes. Airborne imagery proved relevant to acquire simultaneous information on a tree population, notably for characterizing transpiration behaviour at the individual tree scale as a result of images yielded in the thermal infrared domain. Indices derived from high-resolution airborne field imagery appeared to be highly heritable and enabled detection of a large number of QTLs, for vegetation and water stress indices, and the tree response to water deficit. This study opens future avenues for analysis of candidate genes related to foliage response to drought, and may contribute to future selection of new plant woody plant material bred for its response to drought and/or water use efficiency.

## Supplementary data

Supplementary data is available at *JXB* online.

Supplementary Fig. S1. Total QTLs detected on the consensus ‘Starkrimson’ × ‘Granny Smith’ (STK×GS) genetic map.

Supplementary Table S1. Total list of QTLs detected for all phenotypic variables in well-watered and/or water-stress conditions and for the differential index.

Supplementary Table S2. Values of traits (mean and SD) investigated in well-watered or water-stressed conditions and for the differential index.

Supplementary Data

## Abbreviations:

Blup: best linear unbiased predictors
BmFr: fruit yield biomass per tree per year
DI: differential index
G-Blup: Blups extracted per genotype
G-mean: mean extracted per genotype
LAI: leaf area index
NbFr: fruit number per tree per year
NDVI: normalized difference vegetation index
NIR: near-infrared
QTL: quantitative trait locus
RGB: red, green and blue bands
SRPI: simple ratio pigment index
TCSA: trunk cross-sectional area
TIR: thermal infrared
VARI: visible atmospherically resistant index
WDI: water deficit index
WS: water-stressed
WW: well-watered.
