# Supplementary material for: Multispectral airborne imagery in the field reveals genetic determinisms of morphological and transpiration traits of an apple tree hybrid population in response to water deficit
Source: J Exp Bot. 2015 Jul 23;66(18):5453–65. doi: 10.1093/jxb/erv355 (PMC4585425; doi:10.1093/jxb/erv355)
Supplement: Supplementary Data [file supp_66_18_5453__index.html]

Multispectral airborne imagery in the field reveals genetic determinisms of morphological and transpiration traits of an apple tree hybrid population in response to water deficit — Multispectral airborne imagery in the field reveals genetic determinisms of morphological and transpiration traits of an apple tree hybrid population in response to water deficit — Supplementary Data 

# Multispectral airborne imagery in the field reveals genetic determinisms of morphological and transpiration traits of an apple tree hybrid population in response to water deficit

## Supplementary Data

Data files

- Supplementary Data - Supplementary Data
